# Can People Be Managed through Fear? An Enquiry into Arab Culture

**DOI:** 10.3390/bs12100352

**Published:** 2022-09-22

**Authors:** Abdulrahman Essa Al Lily, Ahmed Ali Alhazmi

**Affiliations:** 1 Department of Curriculum and Teaching Methods, College of Education, King Faisal University, Al Ahsa 31982, Saudi Arabia; 2 Department of Education, College of Education, Jazan University, Jazan 45142, Saudi Arabia

**Keywords:** education, technology, sociology, management, anxiety

## Abstract

This research scrutinises the fear-driven management of Arabs. It conceptualises fear as a structural component of Arab society through a sociological lens and, by drawing upon the reflections of 28 Arab experts, philosophises on the potential of fear to develop into a well-configured managerial system (‘feararchy’) that regulates public and private lives. This research finds this system to rest upon a foundation of three forms of fear: ontological (the normalisation of fear), epistemological (the utilisation of fear to shape knowledge) and axiological (the influence of fear on acceptability). This article makes five contributions. First, it shows how, through turning fear into a collective status, Arab managers exploit fear for social control. Second, it demonstrates the institutional nature of Arabs’ fear, making it worthy of attention from the field of management. Third, it investigates fear as an individually internalised, socially constructed feeling that is psychologically manipulative. Fourth, it presents fear as not merely a natural psychological sensation but a managerially distorted contextual frame within which current (and future) members think and operate. Fifth and finally, it exposes the contextuality of fear: sources of fear in one context may not be sources of fear in another.

## 1. Introduction

This research examines the incorporation of fear into Arab organisations under a philosophical microscope. Fear was chosen as the subject of this research to investigate whether fear is the predominant management technique utilised in Arab institutions [1]. The current research is structured around the highly disputed contention that Arabs are managed through fear and is, therefore, enriched by collecting and analysing Arab critics’ thoughts and reflections. These critics were approached through purposeful and snowball sampling and invited to expound on this contention by sharing their thoughts on the management of Arabs through fear. This contention remains unseen and unexamined, as previous attempts to bring it into the light failed in the face of unpleasant and even hostile responses from Arab academia and wider society. Put differently, academics who are willing to speak up about the use of fear will face consequences from their academic peers and society. If an Arab scholar were to prove this contention, they would likely be stigmatised among their colleagues and penalised by their institution, resulting in a damaged reputation, job loss or worse. Consequently, only publications that turn a blind eye to the unhealthy state of Arab society (so called ‘optimistic’) are financially supported. Aware of this political context, the current research intentionally invited Arab scholars with ‘pessimistic’ perspectives to contribute to the study to offset the imbalanced and skewed nature of present-day international literature on Arab society.

## 2. Literature Review

This article addresses components of Arab culture related to the emotional state of fear and examining the manifestations of fear-based management and showing how this managerial style manifests itself in Arab regions. It documents the far-reaching influence of fear in the management of Arabs and philosophises on the potential of fear to develop into a well-configured managerial system (‘feararchy’) that regulates public and private lives. Virilio and Richard [2] agree that fear can be ‘administered’ as an instrument for the preoccupation of social spaces, and this administration can be developed into a ‘ministry of fear’ [3]. Massumi [4] refers to the saturation of day-to-day interaction by fear, and Sari [5] goes a step further by calling the entire twenty-first century a feararchical century. Ibrahim [6] extends this argument to its extreme, seeing the whole world as a feararchical planet. Fear can, as observed by Odeh [7], be constructed as a structural foundation for individual and institutional behaviour. Fear does not merely reflect a natural psychological sensation but can be managerially distorted to function as a contextual frame within which current (and future) members think and operate [8]. As Farhat [9] reasons, fear may be formed to shield cultures and safeguard traditions, permitting the remnants of past fear to frame the present. Fear can, in the eyes of Burkose [10], be utilised to limit social action and, therefore, give a community boundaries of fear.

Existing Arab literature seems essentially skewed to psychological theories, viewing fear predominantly through psychological lenses [11]. Yet, the current research is informed by the field of sociology, analysing fear mainly as a sociological phenomenon. The current study seeks to socialise and philosophise fear, whereas other Arab studies leave the sociological aspect of fear in the shadows [12]. Arab literature views fear as an apolitical matter that lies beyond politics and cannot be subject to external manipulation [13]. However, the present research views fear as a political issue, seeking to draw attention to the political and unethical aspects of fear. Arab literature sees fear as a natural, primitive, individualistic and personally realised feeling, looking at individualistic fear and scrutinising fear as a personal matter [9]. However, the current research regards fear as sometimes being an unnatural, institutionalised feeling that is socially constructed and manufactured, looking at collective fear and scrutinising fear as an institutional topic. Arab academic writers perceive fear as being real and resulting from real threats and dangers [14] while this manuscript perceives fear as, at times, appearing real, even though it is fundamentally illusory and has no real substance. Arab writings view fear as coming from within individuals and as an inner experience [6]. Nevertheless, the current paper views fear as coming from outside individuals and internalised into their inner system. Existing Arab literature regards fear as being rationally justified [15]; the current research, however, regards fear as resisting rationality. Arab literature sees fear as context-free, meaning that individuals fear certain matters and issues despite the context from which they originate [13]. In contrast, the study undertaken here sees fear as context-dependent, meaning that what people fear in one context (e.g., fearing certain ethnicities or religions) may not be a source of fear for people in other contexts.

Arab literature is uncomfortable with the promotion of innovative approaches to fear, refusing to think otherwise and challenge academic configurations and conventions [7]. In comparison, the current research encourages an innovative approach to fear, promoting the unconventional view of fear through a managerial lens and exposing it to the field of management. This literature sees fear as the individual’s attempts to avoid naturally realised danger [5]. By contrast, the current research sees fear as referring to the individual’s attempts to avoid communally and culturally moulded harm. Arab works view fear as being triggered by potential and actual risks, away from political motives [16]. The present work views fear as also being triggered by hatred and other political motives. Arab literature thinks of fear as being driven by the basic human instinct of wanting to survive, protect oneself and one’s family and remain safe [17]. The current publication thinks of fear as also being utilised as a source of power to reinforce norms, control behaviour, drive societies and ensure obedience to social and cultural parameters. Arab literature is unconvinced by the non-positivistic interpretivist nature of a qualitative enquiry, encouraging the quantitative examination of fear [8]. The current research is inspired by the interpretivist nature of a qualitative enquiry, putting to rest the positivistic parameters of quantitative methods. Arab literature shows extensive interest in quantitative generalisation, comparison and causality, for instance, looking at the causes and effects of fear [10]. On the contrary, the current research shows extensive interest in descriptions, looking at the manifestation of fear. Arab literature fears studying fear politically to avoid political trouble [18]. In contrast, the current research takes a political risk and examines the political shaping of fear.

## 3. Methodology

This research is structured around the following research question: in what ways are Arabs managed through fear? This question is supplemented by three sub-questions: (1) in what ways is fear normalised? (2) to what extent is fear utilised to shape knowledge? and (3) in what ways does fear influence what is acceptable? In this work, the authors strive to bring the domination of institutional fear in Arab society to the fore, radically questioning this system of fear-based management, as they see critique and analysis of this domination as tools to deconstruct and dismantle the system. This research is intended as both an academic and political intervention to raise not only the public’s but also academics’ consciousness about the utilisation of fear to control Arab society. A conceptual framework for this study, deemed necessary by the authors, was established by those few Arab academics, journalists and critical users of social media conscious of the use of fear for social control who, through invitation and interview, lent their support to such an intervention. The current manuscript aims to foster a process of depoliticisation, through which individuals are encouraged to see for themselves how their emotional system is manipulated for the sake of politics.

The authors searched for potential interviewees in various domains: academic literature, the journalistic field and social media. Twenty were identified, contacted and asked to recommend others also aware of fear-based control, resulting in an additional 19 possible interviewees. With both purposeful and snowballing sampling techniques, 39 subjects were found for this investigation and invited to participate, of which six declined and five ignored the invitation, resulting in a total of 28 interviewees. Of these participants, all were over the age of 25, 19 were PhD holders, 27 were married, and four were women. This research runs across the Arab world, with these participants from Jordan, Syria and Lebanon in the Levant, the United Arab Emirates, Saudi Arabia and Bahrain in the Arabian Peninsula, and Egypt, Sudan and Tunisia in Africa.

The interviews were designed to be unstructured, encouraging interviewees to speak freely about the fear-based management of Arabs, conducted using a videoconferencing system (namely, Whereby) and lasted more than one hour. The interviews were not recorded to give the interviewees peace of mind, wherein they could express their views openly (and without fear). That said, exhaustive notes were taken during each interview, and a subsequent report was written up. The report was sent to the interviewees to verify its content and clarify misunderstandings. Interviews were not the only data source for this study; documents, including textbooks and policies, were also analysed to comprehend institutional fear.

The norm in Arab academic literature is for authors to idealise, support and sing the praises of Arab society. Criticisms of Arab society, especially by Arabs themselves, are not only undocumented but also suppressed and repressed. As a response to this non-academic condition of the literature, this study was crafted to expose those experiences within Arab regions that are profoundly and purposefully unexplored and unrepresented. Accordingly, a key criterion for inclusion in this study was that the participant was known for speaking against this hegemonic positivity. Politically motivated, this investigation seeks not to reflect Arabs’ conventional, diplomatic views of their own society but rather to explicitly feature critics who are ready and confident to oppose and ‘trouble’ the mainstream. Put differently, destabilisation of this kind, regardless of the outcome, is a goal in itself. Accordingly, the authors solicited critics’ reflections on the managerial status of Arab regions and, through applied snowball sampling, enabled participants to suggest other suitable critics. In this way, interobserver reliability was ensured even before data collection, as the observers were selected specifically for their shared beliefs concerning the existing fear-based management of Arab society and its organisations.

**Figure 1:**
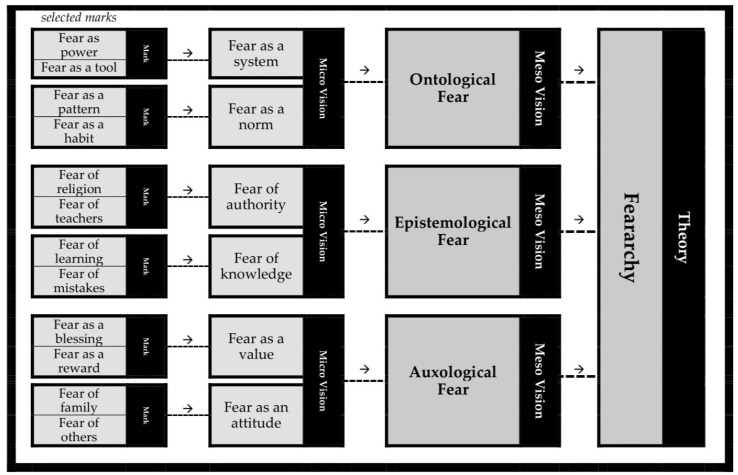
Data Analysis.

This research applied the grounded-theory approach, an academically recognised systematic methodology mostly utilised for qualitative enquiries [19]. This approach involves mainly inductive reasoning, having the power to aid with the systematic discovery and construction of a theory from data [20]. As investigators judiciously scrutinise the data collected, ideas become apparent to them. These ideas are supposed to emerge from the data. The investigators then tag those ideas with marks that capture the core of these ideas. As more data arrive and are inspected, the marks can be grouped first into micro-visions and then into meso-visions. Meso-visions then become the basis for a new theory. Hence, the grounded-theory approach is unlike conventional approaches where researchers select pre-existing theoretical frameworks, develop hypotheses derived from these frameworks and collect data with the aim of checking the validity of these hypotheses [21]. Echoing this reasoning, the collected data were analysed methodically and systematically, guided by the parameters of the grounded-theory approach (see Figure 1). For each interview, the interviewee’s reflections were grouped into distinct meanings, each of which is marked using a Latin numeral; likewise, each interviewee is labelled with an Arabic numeral. Accordingly, we can understand ‘IX:5′ denotes the 9th mark of the 5th participant. Marks of the same type are assembled into micro-visions, and micro-visions with similar meanings into meso-visions, thus building a foundation for the theory. Hence, this fear framework is derived from the research that the authors undertook, taking advantage of the grounded-theory approach. To explain Figure 1, ‘fear of religion’ and ‘fear of teachers’, for instance, were marked. Since these two marks are similar, they were combined, generating a micro-vision entitled ‘fear of authority’. However, this micro-vision is similar to another micro-vision (i.e., ‘fear of knowledge’ that is made of two marks: ‘fear of learning’ and ‘fear of mistakes’). Because of this similarity, these two micro-visions were grouped to generate a meso-vision named ‘epistemological fear’. This meso-vision was grouped with the meso-visions ‘ontological fear’ and ‘auxological fear’ to generate a theory: ‘feararchy’ (i.e., the utilisation of fear as a managerial system).

The sample size of this research can be justified in two ways. First, the research is not intended to make generalisations but rather to build a conceptual framework for a management style (here, fear-based management). Hence, what is essential is not the quantity of interviewees, but rather the quality of interviews, (i.e., how descriptively detailed and analytically exhaustive interviews are) so as to establish a rich conceptual framework. Second, saturation in grounded theory exceeds the concentration upon sample size as the justification of sampling adequacy, given that ‘sample size in grounded theory cannot be determined a priori as it is contingent on the evolving theoretical categories’ ([22] p. 3). That is, the authors noticed that, after the 28th interview, theoretical categories had evolved sufficiently and, therefore, the authors saw no necessity to recruit more interviewees. This quantity of interviews is in agreement with the existing literature, which assumes that a number of 25 to 30 interviews is sufficient for the development of a theory and the establishment of a conceptual framework [23].

This article endeavours to be a philosophical unravelling of the underlying dimensions of feararchy, rather than an empirical contribution to research, for two main reasons. First, because Arab academics tend to avoid engaging in the philosophisation of Arab society, a vacuum is left inevitably filled by Western academics. Hence, this study is a wake-up call to Arab academics, who habitually perceive themselves as ‘empirical researchers’, reminding them to philosophise and develop a philosophical worldview comprised of wholly ‘domestic’, non-imitative theoretical constructs. Second, this research is designed to construct a theory to act as a ‘start-up’ of the parameters on which future studies will be able to rely to empirically enquire into the role of fear in the management of Arab society.

## 4. Findings

Emerging from the data analysis is a clear picture: while the human brain naturally has a database of matters that represent psychological fear, some societies and cultures intervene and ‘hack’ into this database to insert new fear-representing matters (i.e., socially formed fear). As a consequence of such an intervention, the human brain becomes alarmed by matters that are psychologically and naturally non-fearful and even healthy and beneficial. This societal intervention, as evidenced in the data analysis, endeavours to patronise three forms of fear (i.e., meso-visions). The first meso-vision is ontological fear, which normalises fear to be internalised into individuals’ inner worlds and objectified into policies, strategies and practices. The second is epistemological fear, which uses fear to shape the relationship between individuals and knowledge and to control access to knowledge. The third is axiological fear, which transmutes fear into a value and uses it to influence individuals’ understanding of what is acceptable. By drawing upon the insights gained from the interviews, in the following sections, we make an account of meso-visions, micro-visions, and their marks in great detail to build a solid foundation for the macro-vision.

### 4.1. Ontological Fear (Meso-Vision)

In what ways is fear normalised? The answer to this sub-question explicates the meso-vision of ontological fear by laying out the ways in which fear is actualised, standardised and institutionalised and unpacking this meso-vision into its two micro-visions: ‘fear as a system’ and ‘fear as a norm’.

#### 4.1.1. Fear as a System (Micro-Vision)

This micro-vision draws from two marks: ‘fear as power’ and ‘fear as a tool’. A mark is fear as power. When experiencing fear, ‘humans hit their weakest point and become more obedient’ (VI:17); hence, fear is often exploited, as an institutional power and managerial system (‘feararchy’), to ease social control and cultivate submissive mindsets (XX:11). Institutional fear is fear integrated into policies, strategies and practices, that is, ‘making institutions haunted by fear’ (X:6). Forming policies of fear, as noticed by some participants, mutually feeds into and feeds off the action of developing strategies of fear. Similarly, establishing strategies of fear both results in and results from undertaking practices of fear. Expressed differently, ‘the relationship amongst approaches, procedures and actions of fear is dialectic’ (XXVIII:16). Ergo, it is not straightforward to figure out whether institutional fear comes from above or below. Nevertheless, what is easily established is whether or not ‘a whole community [is living] in a position of managerial fear’ (XIV:38). One symptom of an institution managed by fear (‘feararchical’) is that affiliates are at the same time victims and producers of fear, playing a dual role. Fear is embedded into affiliates at all levels, generating a permanent state of fear throughout the institution (‘feardom’). When fear exists as both ‘a norm and part of members’ character’ (XXVIII:32), members ‘live with fear and by fear and possess it as a habit’ ([15] p. 3). This is what is called institutional fear, and it is political, with the power to induce behaviours, justify policies and ‘distract the public’s attention from allegedly more urgent social issues’ ([24] pp. 171–172). Once such fear is normalised, fear itself transforms: from a state of alertness (e.g., to avoid danger) to a lifestyle [25]. In the words of one interviewee, ‘to live in a fear-determined community is to take on a fearing culture’ (XXXIII:4).

A mark is fear as a tool. A straightforwardly one-way political relationship also exists between ‘the fearee’ and ‘the feared’, with the former using their political power to exert fear over the latter. While technological advances have destabilised and unbalanced this relationship, depoliticising feararchical systems by encouraging the feared to be less afraid [18], technology can also be used as a political tool by the fearee for greater social control, implanting even more fear into the lifestyles and mindsets of the feared. In this way, technology is leveraged for the ends of institutional fear, limiting ‘humans, their freedom and their creativity’ ([26] p. 5). In the data, we found three key examples to support this claim. Example 1 concerns the use of CCTV cameras to further institutional fear. In institutions of the feared, a whole budget and committee are often dedicated to the installation and maintenance of such cameras in corridors, which promotes both actual surveillance and the feeling of being watched. This is not to mention the concrete gated fences enclosing educational institutions. Some students reportedly climb these fences during school time to ‘break free’. These students are inevitably punished the day after their escape; one interviewee was drawn to the thought that feararchical schooling ‘functions more like a prison’ (XIII:2). Example 2 concerns the use of devices to further temporal fear. Biometric devices can be used for attendance: an employee late by only one second is still punished by having this second deducted from their salary, depriving them of humanitarian flexibility. Example 3 concerns the use of electronics to further sensory fear. The tone of school bells is designed to sound aggressive and hostile, without any clear reason why it does not sound like the twitter of birds, music or really any tone conveying a sense of friendly attention —not intimidating warning and, thus, institutional fear.

#### 4.1.2. Fear as a Norm (Micro-Vision)

This micro-vision draws from two marks: ‘fear as a pattern’ and ‘fear as a habit’. A mark is fear as a pattern. Once institutional fear reaches its tipping point, it achieves a process of progressive self-generation, that is, ‘it generates itself, making members live in a loop and circle of fear’ (XIX:39). It takes on a life of its own, coming from above, from below and from every direction, supported by managers and members themselves [13]. Brainwashed by fear, members voluntarily accept and choose to be feararchicalised, ‘loving their abusers and determining the Stockholm syndrome’ (VIII:37). A widely exchanged saying is ‘whoever prioritises fear remains safe’, which is coupled nicely with the saying ‘the more one makes oneself fear, the less likely one exposes oneself to punishment’. In this way, once fear is normalised, pathological actions and ‘practices such as distrust, deception and manoeuvring become popular behaviour that one undertakes as a defensive way of running away from day-to-day fearful encounters’ (I:29). For one interviewee, ‘when fear constitutes the norm, illusion, accordingly, constitutes the norm too’ (XXIX:36). As a norm, fear is conveyed not only to members but also between generations, ensuring the continuation of ancestors’ fear into the present [10]. As remnants of past fear move across generations, they become, as some interviewees observed, taken for granted, and fear becomes a heritage, an integral thread in the social fabric, or a silent yet ever-present material reality, within which members perform their day-to-day activities.

A mark is fear as a habit. In a feararchical society, individuals exhibit fear responses—even in the absence of fear stimuli—because being fearful has become a habit. Individuals take pleasure in being fearful and continually alarmed, regarding this as something positive and something that makes them look awake [27]. This outlook turns one’s whole life ‘into a permanent alarm and everlasting concern, […] implicitly affecting one’s mental health, well-being and welfare’ (IX:1). One interviewee is convinced that fear ‘regulates institutions’ vision, mission and values, providing overall direction for everything that occurs in these institutions’ (XXI:7). Institutional fear exists as an integral part of built environments and ‘influences society not randomly but rather in intentional manners’ (XXXI:8). It follows a ‘pedagogy of fear’, referring to the instructional incorporation of fear into an institutional context and its application for regulating the communications that happen within daily organisational life. A feararchical society places the concept of failure at the forefront of individuals’ minds, making them fear life and embrace a survival mentality. Failure is so well incorporated that ‘one’s concept of success is limited to the avoidance of failure, rather than the exploration of opportunities and the celebration of positive outcome’ (XXXIV:20). In textbooks, the entirety of a human life is reduced to a lifelong test, the failure of which members are enduringly supposed to fear. One of the interviewees surmises that failing (and, thus, fear) is always present and at the forefront of members’ minds [6].

### 4.2. Epistemological Fear (Meso-Vision)

To what extent is fear utilised to shape knowledge? The answer to this sub-question explicates the meso-vision of epistemological fear by clarifying how fear is applied to reconfigure the relationship between individuals and knowledge and to control access to knowledge and unpacking this meso-vision into its two micro-visions: fear of authority and fear of knowledge.

#### 4.2.1. Fear and Authorities (Micro-Vision)

This micro-vision comes from three marks: fear of religion, fear of teachers and fear of schooling. A mark is fear of religion. It is the view of advocates of feararchicalism that humans are fearing creatures and, as such, fearing is a value and faith [28]. They transform religiosity from ‘being a source of spirituality, comfort, security and peace to … being a basis of fear’ (XXIV:35). They ‘utilise religion as a fearing tool and cultivate a fear of religion’ (XXI:27). They mispresent the holy power as ‘having the intention to more harm and less reward individuals’ (XIII:34). They ‘give centre stage to the concept of hell, misusing it as an essential tool to make individuals live in a permanent position of fear’ (VII:33). They portray faith as ‘requiring obedience to which one must submit, so as to avoid punishment’ (II:25), while they portray humans as permanently sinful and in need of constantly seeking forgiveness. They are competent at fabricating religiousness to nourish the three following forms of feardom. The first form is fear of music. Feararchicalists cultivate in individuals the belief that, ‘if one listens to music, black lead will be poured into one’s ear in the afterlife’, presenting this belief as sacred, though it is not [29]. The second is fear of others. Feararchicalists falsely refer to certain ‘anti-others’ sayings as divine; one such saying is ‘if one learns the language of others, one becomes aware of their deception’ [29], thereby associating others with deception and elevating the fear of others to include their ethnicities, dialects and languages. The third is fear of women. A common value is that ‘the female voice is provocative and seductive’, making men fearful of hearing women’s voices. This value is popularised as religion, though it is anything but religious in nature. Likewise, feararchicalists present women as deceptive creatures that should be feared by men. There is even a social stigma attached to a man who lacks institutional fear, best illustrated by the fact that a man’s propriety and even religiosity will be brought into question if he does not evince a fear of women.

A mark is fear of teachers. In the land of the fearful, teachers are held in high esteem, even to the extent that a dominant saying is ‘teachers are almost prophets’. However, such high esteem for teachers translates into fear for students, who so fear their teachers that, ‘when they come across [them] on streets, they run away out of subconscious fear even though they are not guilty of anything’ (XXVII:31). One interviewee contends that, in feararchical schooling, ‘teachers have, or at least appear to have, almost unlimited power over students, making students feel fearful about how far teachers may go when punishing them’ (XIX:19). This fear of teachers has evident negative effects on students and their learning; for example, they may be too afraid to participate in classroom discussions or, as one participant deems, they do homework not for the sake of knowledge and cognitive development but because ‘they fear being punished by teachers for not doing homework’ (XV:26). On the first day of school, parents customarily tell teachers the predominant (and yet, ‘deadly fearful’ [III:21]) saying ‘this is our child. Take him in his meat form and return him in his bone form’, with the father granting the teacher permission to harm the child in any way for the sake of education. In feararchical schooling, it is not only students, however, who suffer; it is reported that teachers teach not out of a sense of community service but rather because they fear their headteachers. In this vein, teachers play a dual role: ‘the fearee’ and ‘the feared’, yet it is not only teachers who do so: headteachers, as another participant opines, only do their job because they fear the higher authorities. Feararchy is named thus because it is a hierarchy based on fear; every link in the chain of fear represents another member held captive in a state of fear from above and below.

A mark is fear of schooling. Feararchical schooling boosts and sustains an army mentality. In the data, we found five key examples to support this claim. Example 1 concerns discipline. To enforce militaristic discipline, students begin their day by dressing in uniforms, queuing up for inspection, exercising outdoors—no matter the weather conditions—and walking in orderly queues to their first class of the day. Example 2 concerns naming. In addition to discipline, academic institutions use militaristic jargon to further instil fear and control among students. For example, one student per class is assigned to the role of ‘corporal’ to ‘ensure that his fellow students behave and remain fearful in the absence of their teacher’ (XX:3). Likewise, some feararchical organisations have committees called ‘the High Committee for Punishing Undisciplined Students’. Over and above this, the phrase ‘class management’ is replaced in Arab regions with ‘class controlling’. In institutions of the feared, wording is well articulated to instil fear in individuals: day-to-day discourse, sayings, decisions and writings all overflow with institutional fear-inducing language. Example 3 concerns punishment. Extreme punishments for minor mistakes are another measure schools to enforce fear. For instance, if a student is just a few minutes late, ‘they are punished by making them stand in the sun for hours’ (V:5). Disobedient students may be hit on their palms and legs with sticks. More cruelly, at the beginning of each semester, with a new batch of students to ‘discipline’, the teacher is advised to ‘kill the cat’ (an oft-repeated phrase); the words almost speak for themselves: the teacher punishes one student very severely in order to establish their dominance and instil institutional fear in all other students. Example 4 concerns surveillance. Students’ personal effects and privacy are repeatedly violated by authoritative figures. Feararchical management instils fear in members by frequently checking their mobile phones and auditing their residence to ensure no content is out of line, that is, socially and culturally inappropriate. Example 5 concerns grading. From a feararchicalistic perspective, the truth has been wholly and unerringly preserved in textbooks; obtaining the truth, therefore, is not a matter of interpretation or perspective but of accuracy, which can be tested, assessed and graded, making one’s failure indisputable and inescapable. Each grade, in this view, precisely measures the quantity of the truth one has managed to gain. Receiving a low grade lets down not only one’s parents but one’s entire family or tribe, further worsening the fear of failure.

#### 4.2.2. Fear and Knowledge (Micro-Vision)

This micro-vision draws from three marks: fear of self-exploration, fear of learning and fear of mistakes. A mark is fear of self-exploration. In a fear-normalised society, knowledge is a source of fear, not of exploration. Religious education ‘endorses the idea that knowledge comes from the sky, which humans cannot reach out to but is delivered to them through go-betweens, for instance, prophets, preachers and the like’ (XXVIII:12). Phrased another way, one can access knowledge only through an intermediary, making people fearful of going out into the world and exploring knowledge on their own. As a powerful influence over how individuals think and perceive the world, religious education makes them regard knowledge, be it religious or non-religious, as inaccessible or accessible only through intermediaries, such as teachers, supervisors, advisors, medical doctors and trainers. This implies that the ‘self-exploration of knowledge is not part of these societies and is a cause of fear’ (VII:9). Moreover, despite digital technology maximising accessibility to knowledge as never before seen, members of feararchical societies do not take advantage of this and continue to expect someone superior to deliver and serve knowledge to them. In other words, self-learning is not part of a feararchical culture. Given that digital technology constitutes a potential threat to feararchical societies, it is banned, whether totally or partially, for instance, through ‘a well-designed and well-sponsored system that filters out certain information that is available on the Internet, making members fear this type of information’ (VI:13). Nevertheless, individuals in feararchical societies who believe in free access to knowledge can use techniques such as VPNs to access this ‘filtered-out’ content, in an act that rebalances the power relationship between the fearee and the feared. As an instrument of the fearee to strengthen the feararchical nature of their communities and a means to push back against such social control, ‘technology is both a curse and blessing’ (XXI:31).

Moreover, members perceive life as a source of fear and, thus, danger. They do not see life as an opportunity to explore but rather as a territory infused with explosives to avoid and enemies to run away from. To illustrate, they view job hunting not as an opportunity for growth and success but as a necessary but fearful stop, from which they wish to board the next train as soon as possible. One interviewee claimed that, in feararchical schooling, ‘hope and optimism have no space in the ways through which members see life’ (XXXII:28). Members live as sad and fearing creatures with no concept of fun and entertainment; ‘for fun’, in their eyes, is something that comes from ‘the devil’. A widely reciprocated saying is ‘youth, free time and wealth corrupt individuals dreadfully’, bringing to light how opportunities (here, youth, free time and wealth) are turned by feararchical schooling into fearful hazards.

A mark is fear of learning. Feararchical schooling is beset with standardisation, systematisation, examination and restriction. For instance, it is typical for four exam invigilators to be assigned to supervise a room of 40 students; their ever-presence and determination ‘to act particularly unfriendly … make [sure] students fear cheating’ (XIV:10). Students have to show their ID during the exam to confirm their identity; if they do not bring it, they are punished, even if their own teacher can verify their identity. If students need to visit the bathroom, ‘they are accompanied by teachers to the restroom so as to ensure that they do not cheat’ (XII:14). The committee responsible for organising and managing the exam period is called ‘the Control Committee’; such use of the term ‘control’ instils fear in the hearts of students.

Moreover, individuals are encouraged to learn not for the sake of learning but to avoid the social stigma of being ignorant. A child must bear the demeaning label of ‘the ignorant’, and, for the illiterate adult, such labelling means that they are portrayed as stuck in childhood and unable to mature and embrace adulthood. In this way, ‘one is encouraged to learn out of fear’ (XI:18). A widespread perception is that one learns to avoid evil (or ignorance), not that one learns to gain good (or knowledge). An additional cultural rule is that there is a minimum quantity of knowledge that one is punished for not gaining, making one learn out of fear, not to gain this minimality. ‘I learn because I fear’ forms the essence of feararchical schooling. One participant argues that members are criminalised for thinking for themselves or thinking critically. Put simply, whereas education is, theoretically, supposed to nurture critical thinking, feararchical schooling turns education into a ‘dis-educational’ system, which punishes criticality and, moreover, creativity and giftedness.

A mark is fear of mistakes. During participation in class activities, the simple making of a mistake fates ‘learners [to be] punished bodily or psychologically, not least … shamed in front of other students’ (XXIII:15). Due to this feararchicalistic attitude towards mistakes, practices such as apprenticeship and learning by doing (a way of learning through one’s mistakes) are regarded by members with fear and are, therefore, not part of feararchicalistic culture. Instead, members go for the safest methods of learning, given that such methods as being passively taught and learning through memorisation involve fewer mistakes and, consequently, less fear. When one makes a mistake, ‘punishment is severe and unethical, whether physically or psychologically, via symbolic and verbal violence towards the mistaken’ (XIV:3).

In feararchical organisations, the ‘walls have reporting ears’, as a well-cited phrase goes, capturing the hesitancy of members to express themselves in an environment of institutional fear. This fear of expression has a gendered dimension, deeply affecting women, who are ‘ashamed of publishing and circumvent this shameful practice by writing using pen names’ (XV:22). Nonetheless, the ramifications are society-wide, as discussions become ineffective cognitive tools in a feararchical society because members fear to express themselves freely and genuinely engage in discourse. Members of feararchical communities are held emotionally captive and stuck in a defensive position, ‘attempting to defend themselves against the influx of socially and traditionally perceived fear in their daily life’ (XXV:23).

In a climate of fear, if exposed to criticism, one reacts out of fear and insecurity, defensively pushing back against the criticism to avoid any possibility or potentiality of being punished. One interviewee expressed that a deficit of motivation, coupled with the intensity of institutional fear, discourages members from taking risks and the initiative and, therefore, from moving oneself and the whole community forward. As a corollary, members of these communities tend to be risk-averse, echoing the familiar proverb ‘any door that brings you wind, just shut it and enjoy peace of mind’. They avoid taking the initiative because they see the act as inviting more wind, so to speak. As a result of their permanent feardom, they, as remarked by one interviewee, ‘trust no one and regard others as a source of fear’ (XXXV:12) and repeat the saying ‘beware of your enemy once and beware of your friend a thousand times’. If one is institutionally fearless, one is criticised and perceived as reckless.

### 4.3. Axiological Fear (Meso-Vision)

In what ways does fear influence what is acceptable? The answer to this sub-question explicates the meso-vision of axiological fear by bringing into focus how fear evolves into a value and is used to influence acceptability and unpacking this meso-vision into its two micro-visions: fear as a value and fear as an attitude.

#### 4.3.1. Fear as a Value (Micro-Vision)

This micro-vision draws from two marks: fear as a blessing and fear as a reward. A mark is fear as a blessing. An alarmist view is that humans, feararchically speaking, ‘behave only in the presence and reinforcement of fear, calling for managerial systems that are primarily configured around fear’ (XXXVI:24). Fear is not a sign of something wrong but rather, as feararchicalists would have us believe, ‘a healthy way of taming the wild essence of humans’ (XXVII:23), who will ‘misbehave if [they] know of no subsequent punishment’ (Arabic saying). Fear is thus necessary, for, in its absence, humans will ‘have no concept of goodness, [be] savage and do everything to one another, but nothing good’ (XXXI:25). In the eyes of feararchicalists, ‘humans are naturally disgusting and egotistical unless fear is imposed upon them to control and tame their essential wickedness’ (XXIII:19). In other words, humans need to permanently fear punishment because, otherwise, they will unveil their true nature, which, according to feararchicalists, is violent and destructive. Feararchicalists fear the institutionally fearless, as such humans are embracing ‘their damaging, cruel and insensitive side’ (XXX:20). Moreover, feararchicalists fear humans themselves; after all, in their worldview, ‘war is innate to the human species’ (XXVIII:1), ‘revenge is something that makes people extraordinary’ (XIX:22), and ‘human creatures have no choice but to pollute the environment and be killers of other creatures’ (XXIV:5). Therefore, it is no surprise that, according to feararchicalists, ‘throughout history, human society has become progressively more violent and experienced a gradual increase in the overall degree of brutality’ (XIV:5).

For feararchicalists, humans are not only innately violent; at their core, they are evil; ‘they are the only organisms capable of elaborate lying and of covering their tracks and concealing evidence’ (XXXI:4). Moreover, in a fearless and, therefore, dog-eat-dog world, ‘individuals would have unrestricted desire to do evil and express their full wish for malevolence’ (XXIV:39). Another interviewee made reference to the Arabic proverb ‘an adulteress would like all other women to experience the hell that she endures’ to highlight the feararchicalistic view that a human being ‘has the evil tendency to want other human beings to share the pain that they experience’ (VI:1). It is most evident that the faith of feararchicalists lies in ‘dark philosophy’, a philosophy that only sees tragedy and ascribes such tragedy to human nature; accordingly, a strategy is needed to manage the dark nature of humans, and fear represents an ideal such strategy. Feararchical societies do not conceal their methods of social control but rather, proudly and explicitly, reinforce fear at the same time as they are driven by fear.

A mark is fear as a reward. Feararchical schooling uses fear (i.e., the ‘stick’) over motivation (i.e., the ‘carrot’) to induce socially and culturally desired behaviours, overturning the carrot-and-stick principle. Fear-oriented pedagogies are seen to have the advantages of being ‘more effective, more enduring, less expensive and easily implemented’ (XXXV:8). Nevertheless, because schooling focuses on the stick, rather than the carrot, members lack motivation and only do the bare minimum. Educational institutions committed to developing students’ personal identities through self-confidence bear little resemblance to feararchical schooling, which seeks to fearify members, encouraging them to revert to a self-protection mode, and dysfunctionalise their psychological qualities. In feararchical schooling, considerable effort is made to crush any psychological concept (here, self-esteem or hope) that could challenge the feararchical nature of society. After such schooling, institutional fear continues to closely follow and ‘chase individuals’ [30: 172]; all members of feararchical communities develop the mind and soul of the feared and feel encircled by triggers of fear. In other words, fear has become their fated companion and ‘permanent friend’ (XXXVI:20).

#### 4.3.2. Fear as an Attitude (Micro-Vision)

This micro-vision draws from three marks: fear of family, fear of others and fear of art. A mark is fear of family. Fear-dominated communities preserve a fear of family, namely a fear of one’s parents and a fear of dishonouring one’s family. One is expected to respect and obey one’s parents: the father expects their children to sit, speak and laugh in a particular manner in his presence, and neither parent tolerates any form of confrontation from their children. Parents are not sources of comfort but, instead, of fear, as children whose parents hit them for misbehaving well know. One is to also fear for family, namely one’s family honour, which is a highly respected value and is, notably, associated with the female members of the family in particular. Certain actions and behaviours are to be avoided by all, but especially by women. A woman’s misconduct, however minor, can harm the honour and reputation of the whole family, potentially making not only her but all her male family members unmarriageable. As a consequence, male family members fear for female family members, resulting in overprotective behaviour by men to prevent women’s ‘misbehaviour’.

A mark is fear of others. The fear of others takes two forms in a feararchical society: fear of other cultures and fear of the other gender. The first form, that is, fear of ‘the different’ or ‘the stranger’, is so well engrained that travelling and the Internet, some or all of its contents, can be made culturally inappropriate or even illegal. Different schools of thought are repeatedly described in textbooks as ‘destructive’. Feararchical schooling implants ‘conspiracy’ ([26] p. 28) and ‘cold-war’ (XXVIII:20) mentalities, constantly attempting to convince members that others want to harm them and their culture. Notwithstanding the monocultural and ethnocentric nature of feararchical communities, digital technology has the potential to empower and enable members to open up to others, which would be devastating to feararchicalism. The second form, that is, fear of the other gender, aligns with the broadly accepted belief that, ‘when a man and woman are alone, the devil is the third companion’, promoting disharmony between the two genders [26]. To strengthen this disharmony, feararchical organisations prevent cross-gender friendships (be they offline or online), colleagueships, non-marital relationships (be they romantic or non-romantic) and work-related events to which colleagues bring their partners. All of this is, from a feararchicalistic perspective, considered necessary because women are ‘jewels’ that need to be sheltered so as not to be stolen by ‘thieves’ (men), or women are ‘sheep’ that should fear ‘wolves’ (men). Because of the well-sponsored fear-based relationship between the two genders, women ‘never accept sharing with an unrelated man even a four-seat table on the train, sit next to an unrelated man in cinemas or pass closely by an unrelated man’ (XII:38).

A mark is fear of art. A feararchical society further implants institutional fear into the arts. Perceptions are turned upside down to support feararchicalisation: what was once beautiful becomes fearful. In the data, we found four key examples to support this claim. Example 1 concerns clothing. Dress codes are enforced to instil a sense of fear, for example, if individuals are not dressed in traditionally defined precise ways, they are vulnerable to punishment, humiliation, shaming and social exclusion. Fashion is converted into a dress code: from a source of beauty to a source of fear. If individuals are not dressed in traditionally defined and precise ways, they are vulnerable to punishment, humiliation, shaming and social exclusion. One participant remarked that, ‘even in higher education institutions, there can be a precise dress code for students, support staff and staff’ (XXXIII:28). Moreover, ‘junior managers seek to follow the same style of clothing and shaving as senior managers, fearing punishment if they act differently in terms of clothing and shaving’ (XXXIV:28). Example 2 concerns visual art. Feararchical Arab society eschews any photos, statues, mannequins and dolls in the likeness of humans because of teachings against human creation attempting to simulate God’s creation. Thus, heads are either grotesquely cut off or tastelessly crossed out. Example 3 concerns music. Music is another domain in which feararchicalist societies use and control. They teach people that music and dancing are immoral, contradict spirituality and prevent one from real happiness, as the lyrics and rhythms are so memorable that they distract from spiritual matters. Given that music is disallowed in public, feararchically disobedient members use headsets to listen to music. In doing so, they are taking advantage of the liberative potential of technology, going under the feararchicalistic radar and bypassing societal rules.

## 5. Concluding Remarks

This article has shed light on the regressive features of Arab fear-based management. It has discovered various types of this fear, which are outlined in Table 1. Because of the word limit, components of fear-based management have not been discussed in the current article. One such component is the connection between fear-based management and democratic institutions and civil rights. It would be beneficial if other researchers took up the task of theorising this connection. The most alarming and unexpected finding from this research is that while education is, utopianly, to free humans from unjustified institutional fear, Arab society has used and exploited it for its own advancements at the expense of members’ sense of safety and freedom (see [1,30]). Despite the seeming contradiction, this should not come as a surprise, given that schooling is, after all, a means of ‘forming’ humans and that fear can be employed as a strategy for the successful and effective achievement of this formation. Educational institutions can and do exist as ‘both an expression of a political situation and a teacher of politics [here, fear]’ ([24] p. 65). Arab schooling, as demonstrated in this research, is a domain in which fear is explicitly and implicitly implanted into individuals [30]. Schooling is utilised to foster a culture of institutional fear and, in so doing, facilitate greater social control. While organisations should ideally be led through ‘power and love’ [31], many Arab educational institutions are managed through power and fear.

This article has elucidated the characteristics of Arab fear-based management. It should be said, however, that fear-oriented management does not necessarily exist in all Arab institutions. Instead, it merely constitutes a management style and managerial culture that exist in some Arab countries and organisations and yet are absent in other Arab countries and organisations. Put simply, not all Arab organisations are subject to the influence of fear. There could be a countervailing love through which some Arab institutions are managed (see [32,33]). Love-driven management lies beyond the scope of this research and is a counter management style and management culture that are worthy of academic investigation [34]. However, what the current study shows is that managing through fear normally entails managing through limited love and positive emotion, for example, lacking a thank-you culture. Some directors manage through power and authority, not through positive emotion, meaning that their approach lacks emotional expression. They, for instance, view that the more thanks managers give, the more their public image and power are jeopardised. For them, giving thanks is an ‘employees’ tool to display subordination’ (II:10), implying that, if superiors give thanks to their inferiors, this means reversing the existing power relations and weakening their superiority. Thanking is seen as a symbol of indicating obedience, which inferiors should apply and superiors should avoid, given that ‘glory is the trait of managers, and submission is the trait of employees’ (III:19). The existing managerial culture, furthermore, entails ‘invisible employees’ (XIX:1), a term applied to foreigners performing ‘dirty work’ (XX:35) such as cleaning toilet facilities. These foreigners are sometimes not only not thanked, but even ‘not recognised as existing beings’ (LXX:10). Interviewees described these workers as located ‘below the below’ (LXIX:2) or as ‘third-class members of institutions’ (XXIX:19). An alarmist view in the data suggests that the more powerful an individual becomes, the more likely they are to be less thankful towards their subordinates. An implication is that, if managers were to obtain unconditional freedom and power over inferiors, they might ‘expose their inconsiderate, brutal and destructive darkest side’ (XCIII:33). Other interviewees stated that such managers might ‘have no concept of goodness, not knowing virtue and being vicious, egotistical and wicked’ (LXXV:1) and might ‘perceive themselves as the singular proprietor of the entire universe, not doing their inferiors services and denying their basic rights’ (XVIII:9). This is coupled with ‘making their organisations emotionally malicious and brutish, evil and repulsive’ (XLI:3). One interviewee noted that, ‘being modest, humbled and, thus, thankful is not an option for many managers’ (LXX:16), while another complained that, ‘when one makes an achievement and, therefore, expects thanks from managers, one may rather get blamed or even punished by them’ (XCV:28), demonstrating an extreme form of emotional stinginess. Managers may rarely smile, believing smiling to ‘undermine prestige and go against managerial firmness’ (LIV:11). The lack of thanks given by some managers to employees has been normalised and even institutionalised as good practice in some institutions, meaning that managers who thank their employees are perceived negatively in terms of their managerial awareness and style. Some managers do not thank their subordinates because they want to maintain an emotional ‘power distance’ [35] from them. They are worried that expressions of thanks can shorten this distance and make their subordinates feel overly comfortable with them. Some interviewees subscribe to this belief that the relationship between managers and employees is normally characterised by a high level of formality, thus discouraging managers from thanking employees. In this case, giving praise or thanks is seen to undermine formality.

This article has shed light on the regressive features of Arab fear-based management. It should be said, however, that fear-oriented management is not necessarily an exclusive feature of Arab culture. Rather, it is merely a management style and managerial culture that can exist beyond the Arab context. In this case, Arab culture is not alone in possessing elements and having institutions that exercise fear-driven management. Put in another way, fear-driven management is not a distinction between the Arab and the non-Arab domains. For instance, a reading of the Western literature can demonstrate how previous eras of the West (and, moreover, some current Western societies) share various aspects of Arab fear-based management (see, for example, [36]). One example is that the discussed pathological status of Arab academic institutions (where members resort to chronic actions of compulsive or habitual lying as a survival tactic against fear-based management) can be argued to occur in the academia of some Western countries. Another example is that the role of religious teachings in promoting fear in Arab countries could be claimed to be similar to that in past Western civilisations. Besides, the debated fear-based relationship between the genders can be claimed to be present in some present-day Western societies. Similarly, the explained fear of schooling (or what could be called ‘school complex’) is a problem that many modern Westerners have in common with some Arab regions. Likewise, likenesses could be assumed to exist between Arab countries and some Western countries in terms of a fear-driven ‘trust complex’ where one cannot trust other humans and, moreover, regard them as a cause of horror and distress. By the same token, ‘fear-driven relationship’ between the genders is a dilemma not only in Arab cultures but across countries and cultures. It can be argued that there is a limited essential difference in terms of the fear of the other gender concept in both regressive and progressive cultural contexts. Bearing these examples in mind, it would be beneficial if articles (at least commentary articles) were written which discuss the findings of the current manuscript in relation to other current and past non-Arab communities and civilisations and to point out the degree to which these other communities and civilisations exercise or have exercised fear-determined management. The regressive culture of fear from a historical perspective, delivering more historical insights concerning the evolutions of habits, norms and culture is worthy of further academic investigation.

The target groups of this study are anyone who has been subject to any form of fear, i.e., who have been managed through fear. That said, one may argue that all humans have at some point in their life managed through fear and encountered fear-based management whether in small, moderate or extreme quantities. A further argument could be that humans spontaneously play two roles, at times and in certain situations being the fearee and at other times and in other situations being the fearer. The acts of both exercising fear on others and being a victim to fear can be assumed to be the norm in human society. To take it to the extreme, it could be argued that, in all various human (and perhaps non-human) societies, fear-oriented management, inevitably, existed in their past, exists in their present and will exist in their future, but to different degrees. The intensity of fear-oriented management can be high in one country and yet low in another. Some societies are worse and more explicit than others when it comes to administration through fear. The total elimination of fear-driven management could be claimed to be merely a utopian concept. To link this article to recent global events, it could be reasoned that the COVID-19 pandemic is an appropriate occasion for managerial reforms that are intended to lessen the elevated intensity of fear in some workplaces, given the very obvious role that the pandemic has played in deeply reconfiguring many well-established norms, regulations, values and practices despite the disapproval of employers. Working from home has remarkably reduced the control (and, thus, the fear) that employers may exert over employees.

## Figures and Tables

**Table 1:** The Effects of Fear on Individuals and Institutions.

Type of Fear	The Effected	Effort
Fear as power	Individuals	Fear as power makes individuals more obedient, as they are afraid of the consequences of acting otherwise.
	Institutions	Fear as power makes institutions less likely to experience bottom-up improvements, as members are afraid of the consequences of disturbing the top-down nature of hierarchies through the suggestion of improvements.
Fear as a tool	Individuals	Fear as a tool exploits any medium (be it digital or non-digital) to sponsor the intention to exercise fear over individuals.
	Institutions	Fear as a tool exploits any possible medium to strengthen fear-based management, thus making the entire institution live in a state of fear.
Fear as a pattern	Individuals	Once fear is normalised, pathological actions and practices such as distrust, deception and manoeuvring become popular behaviour that one undertakes as a defensive way of running away from day-to-day fearful encounters.
	Institutions	Once institutional fear reaches its tipping point, it achieves a process of progressive self-generation, becoming a silent reality that is conveyed across generations.
Fear as a habit	Individuals	Once being fearful becomes a habit, individuals exhibit fear responses even in the absence of fear stimuli.
	Institutions	One fear becomes an institutional habit, it regulates institutions’ visions, missions and values, providing overall direction for everything that occurs in these institutions.
Fear of religion	Individuals	Humans are portrayed as permanently sinful and, therefore, in need of constantly seeking forgiveness, thus keeping individuals in an enduring state of guilt.
	Institutions	Religion is transformed from being a source of spirituality, comfort, security and peace to being an institution of fear.
Fear of teachers	Individuals	Students are too afraid to participate in classroom discussions and do homework not for the sake of knowledge and cognitive development but because they fear being punished by teachers for not doing homework.
	Institutions	Teachers are seen not as a source of enlightenment, but rather as a source of discipline.
Fear of schooling	Individuals	In institutions of the feared, wording is well articulated to instil fear in individuals.
	Institutions	Schooling is preserved as a place of being evaluated, tested and judged, not as a place of mental and motor development and discovery.
Fear of self-exploration	Individuals	Individuals are brought up to believe that access to knowledge can take place merely through an intermediary, making people fearful of going out into the world and learning on their own.
	Institutions	Fear-oriented institutions do not enable members to explore and suggest alternative ways of improving their institutions.
Fear of learning	Individuals	Individuals are encouraged to learn not for the sake of learning but to avoid the social stigma of being ignorant, i.e., learning out of fear.
	Institutions	Learning is represented as being at times bad; hence, the authorities regulate what individuals should and should not learn, making them watch out for what they learn.
Fear of mistakes	Individuals	Owing to the fear of mistakes, practices such as apprenticeship and learning by doing (a way of learning through one’s mistakes) are not part of fear-oriented institutions.
	Institutions	The simple making of a mistake fates one to be punished bodily or psychologically, discouraging them from engaging in any form of participation, taking risks or initiatives and, therefore, from moving oneself and the whole community forward.
Fear of others	Individuals	Fear-oriented societies implant conspiracy and cold-war mentalities, constantly attempting to convince members that others want to harm them and their culture.

## Data Availability

The data are confidential and, therefore, not available to the public.
